# *Mycoplasma genitalium* infection in the female reproductive system: Diseases and treatment

**DOI:** 10.3389/fmicb.2023.1098276

**Published:** 2023-02-21

**Authors:** Jianwei Yu, Yan Zhou, Haodang Luo, Xiaoling Su, Tian Gan, Jingyun Wang, Zufeng Ye, Zhongliang Deng, Jun He

**Affiliations:** ^1^Department of Public Health Laboratory Sciences, School of Public Health, Hengyang Medical School, University of South China, Hengyang, Hunan, China; ^2^The Affiliated Nanhua Hospital, Department of Clinical Laboratory, Hengyang Medical School, University of South China, Hengyang, China

**Keywords:** *Mycoplasma genitalium*, female reproductive diseases, inflammation, tumor, treatment

## Abstract

*Mycoplasma genitalium* is a newly emerged sexually transmitted disease pathogen and an independent risk factor for female cervicitis and pelvic inflammatory disease. The clinical symptoms caused by *M. genitalium* infection are mild and easily ignored. If left untreated, *M. genitalium* can grow along the reproductive tract and cause salpingitis, leading to infertility and ectopic pregnancy. Additionally, *M. genitalium* infection in late pregnancy can increase the incidence of preterm birth. *M. genitalium* infections are often accompanied by co-infection with other sexually transmitted pathogens (*Chlamydia trachomatis*, *Neisseria gonorrhoeae*, and *Trichomonas vaginalis*) and viral infections (Human Papilloma Virus and Human Immunodeficiency Virus). A recent study suggested that *M. genitalium* plays a role in tumor development in the female reproductive system. However, few studies endorsed this finding. In recent years, *M. genitalium* has evolved into a new “superbug” due to the emergence of macrolide-and fluoroquinolone-resistant strains leading to frequent therapy failures. This review summarizes the pathogenic characteristics of *M. genitalium* and the female reproductive diseases caused by *M. genitalium* (cervicitis, pelvic inflammatory disease, ectopic pregnancy, infertility, premature birth, co-infection, reproductive tumors, etc.), as well as its potential relationship with reproductive tumors and clinical treatment.

## 1. Introduction

In 1981, Tully et al. (1981) first successfully isolated two strains of *Mycoplasma genitalium* (G-37 and M-30) from 13 male patients with non-gonococcal urethritis. With the development of culture technology and detection methods, *M. genitalium*, a newly emerged sexually transmitted disease pathogen, has received increased attention. According to the 2021 guidelines for the Sexually Transmitted Infections Treatment issued by the United States Centers for Disease Control and Prevention, *M. genitalium* infection can cause cervicitis and pelvic inflammatory disease (PID) and more evidence is needed to determine whether *M. genitalium* infection is associated with adverse pregnancy outcomes (Workowski et al., 2021). A meta-analysis showed that *M. genitalium* infection was significantly associated with an increased risk of cervicitis (pooled odds ratio [OR] 1.66), pelvic inflammatory disease (pooled OR 2.14), female infertility (pooled OR 2.43), preterm birth (pooled OR 1.89), and spontaneous abortion (pooled OR 1.82; Lis et al., 2015). A recent meta-analysis from China showed that the infection rate of *M. genitalium* infection in pregnant women was 4.86%, and the *M. genitalium* infection rates in women with ectopic pregnancy, spontaneous abortion, induced abortion, and premature rupture of membranes were 13.01, 11.81, 6.11, and 12.63%, respectively (Yan et al., 2022). This suggests that the incidence of *M. genitalium* infection in pregnant women with ectopic pregnancy, spontaneous abortion, and premature rupture of membranes is higher than that in other pregnant women. Therefore, it is necessary to pay attention to the related diseases and treatment of *M. genitalium*.

Routine screening for sexually transmitted infections (STIs) usually targets only *Chlamydia trachomatis* and *Neisseria gonorrhoeae,* the two most common bacterial STIs globally, whereas *M. genitalium* infection is an emerging STI with symptoms similar to these two infections. Studies on the prevalence of *M. genitalium* infection in women indicated that *M. genitalium* infection is often co-infected with other sexually transmitted pathogens, including *C. trachomatis*, *Trichomonas vaginalis,* and *N. gonorrhoeae*. Co-infection of *M. genitalium* with *C. trachomatis* was reported at 29.9% and co-infection of *M. genitalium* with *N. gonorrhoeae* at 23.6% in young women at high risk in the United States (Sena et al., 2018), while in a study of testing centers in Belgium, Germany, Spain, and the United Kingdom, the rate of co-infection of *M. genitalium* with *C. trachomatis*/*N. gonorrhoeae* was only 0.1 to 0.6% (Perry et al., 2022). It is unclear whether this difference is related to the underrepresentation of the collected samples and further investigation is needed. *C. trachomatis* is the most common pathogen co-infected with *M. genitalium*. Most importantly, the detection rate of *M. genitalium* in high-risk populations is higher than that of *C. trachomatis*, *T. vaginalis*, and *N. gonorrhoeae* in some studies (Sena et al., 2018; Trent et al., 2018; Nolskog et al., 2019). The 2015 French guidelines for diagnosing and treating PID have included *M. genitalium*, *N. gonorrhoeae,* and *C. trachomatis* as routine targets of microbial examination for PID (Brun et al., 2016).

In addition to causing inflammation, many studies showed that persistent exposure to other Mycoplasma species is associated with oncogenic transformation and leads to proliferation, invasiveness, and metastasis of cancer cells (Zarei et al., 2013; Gedye et al., 2016; Zella and Gallo, 2021). Recent studies suggested that *M. genitalium* is associated with ovarian cancer (Fortner et al., 2019) and high-risk Human Papilloma Virus (HPV) infection (Ye et al., 2018). These findings indicate that *M. genitalium* adversely affects the health of the female reproductive system. Hence, this article reviews the diseases of the female reproductive system caused by *M. genitalium* and its treatment.

## 2. Molecular pathogenic characteristics of *Mycoplasma genitalium*

*Mycoplasma genitalium* harbors the smallest genome among self-replicating prokaryotic cells and lacks a cell wall. It is estimated that the genome contains about 500 genes, including guanine - cytosine average content is shallow, about 31% (McGowin and Totten, 2017). Lacking almost all enzymes required for amino acid biosynthesis, *de novo* nucleic acid synthesis, and fatty acid biosynthesis, *M. genitalium* shows marked metabolic limitations and can metabolize glucose but not arginine or urea. *M. genitalium*, is thus dependent on the host’s nutrients and grows slowly in an SP-4 medium rich in amino acids, nucleotides, glucose, vitamins, and cholesterol. In the absence of a cell wall, *M. genitalium* encodes a large number of lipid-associated membrane protein (LAMPs) genes (Fraser et al., 1995), which may be in prolonged contact with the epithelial surface during acute and chronic infection. These LAMPs play a role in innate immune activation of epithelial and resident immune cells by binding to highly expressed pattern recognition receptors, ultimately leading to activation of cell-host defense pathways, secretion of pro-inflammatory cytokines, and continuation of local inflammatory responses (Dehon and McGowin, 2017).

*MgpB* and *MgpC* are major genes involved in the genetic diversity of *M. genitalium*. *MgpB* (also known as MG_191) encodes the MgPa protein (also known as P140), which mediates adhesion to cell types such as human oviduct hairy epithelial cells. *MgpC* (also known as MG_192) encodes the P110 protein (also known as P114). *In vivo*, *MgpC* heterogeneity is extensive and evolves throughout for persistent infection, with sequence variation within *MgpC* supporting recombination with MgPar regions. MgPar is a general term for the nine repetitive DNA sequences that compose the chromosome of *M. genitalium*, and contains 79–90% of *MgpB* and *MgpC* gene homology sequences (Fraser et al., 1995). Since MgPar sequences have only partial and incomplete copies of *MgpB* and/or *MgpC*, which are homologous to different regions of these two genes, such homologous but not identical MgPar sequences are recombined into *MgpB* or *MgpC*, resulting in the expression of variant MgPa and P110 proteins, causing genome heterogeneity of *M. genitalium* (Iverson-Cabral et al., 2007; Hakim et al., 2021). The antigenic variation of these proteins can not only optimize adhesion, but also help to avoid host immune response and promote persistence, which plays a key role in the pathogenicity of *M. genitalium* and escape from the host immune system, and is essential for the survival of *M. genitalium* (Taylor-Robinson and Jensen, 2011). Genotyping revealed that the variety of *MgpC* repeat regions occurred in a single infected strain, suggesting that the antigenic variation of *M. genitalium* could be achieved by recombining between different sites of the MgPar sequence and recombining of *MgpB*, *MgpC*, and MgPar (Iverson-Cabral et al., 2007). In addition, *MgpB* genotyping has significant genetic diversity in different populations of patients, so it is necessary to carry out molecular typing of *M. genitalium*. Currently, *M. genitalium* strain typing is mainly based on analyzing different short tandem repeats of *MgpB* alleles MG_191 and MG_309 (Pineiro et al., 2019; Laumen et al., 2021; Dumke, 2022).

*Mycoplasma genitalium* morphologically presents as a bottle or flask shape, with a rod-like structure at the end, and a tiny protrusion (7–8 nm) similar to the protrusion of myxoviruses, which can promote adhesion and make it have adhesion force. The terminal structure is composed of various proteins, among which P140 and P110 are the major adhesion proteins of *M. genitalium* (Hu et al., 1987), which need to cooperate with accessory proteins. The adhesion of P140 protein is an essential step in producing pathogenic changes (Taylor-Robinson and Jensen, 2011). It is not only an important functional component of organelles but also the strongest immunogen according to human and experimental animal models (McGowin et al., 2013). P110 is an immunodominant protein of *M. genitalium*, and its binding to sialic acid oligosaccharides activates the adhesion of *M. genitalium* to human cells (Aparicio et al., 2018). P140 and P110 interact to form a transmembrane complex called “Nap,” which accumulates at polar terminal structures and plays an important role in cell adhesion and motility (Aparicio et al., 2020). In addition, the biofilms generated by *M. genitalium in vitro* are rich in polysaccharides composed of poly-N-acetylglucosamine (PNAG), which can reduce contact with antibiotics and increase the resistance of *M. genitalium* (Daubenspeck et al., 2020).

The above characteristics of *M. genitalium* justify its pathogenicity at the molecular biological level. It is not known whether the dependence of *M. genitalium* on its environment is related to its selectivity and persistence in the infected host. For patients with multidrug-resistant *M. genitalium* infection, it is necessary to determine whether microecological regulation of infection facilitates pathogen clearance.

## 3. *Mycoplasma genitalium* and the female reproductive system

The incidence of *M. genitalium* infection varies greatly among different ages, regions, and populations. Studies demonstrated that the infection rate of females aged 15–21 was higher than that of females aged 22–25 (22.6 and 17.7%, respectively; Lillis et al., 2019). In a retrospective study from 2009 to 2019, the detection rate of *M. genitalium* was 3.4% among 17,573 women who presented a pregnancy termination and contraception clinic, with the highest prevalence of 5.4% among 20–24 years. The prevalence fluctuated over time, from 4.4% in 2009 to 2.1% in 2013 and 4.8% in 2019 (Shilling et al., 2022). A meta-analysis revealed that the prevalence of *M. genitalium* in the general population of developed countries was 1.3%, lower than 3.9% in underdeveloped countries, and the prevalence of sex workers was as high as 15.9% (Baumann et al., 2018). A sample of female sex workers from Burkina Faso showed an infection rate of 11.54% of *M. genitalium* (Tovo et al., 2021). The frequencies of *M. genitalium* in the New Orleans sexually transmitted disease clinics (Lillis et al., 2019), a multicenter clinical study in the United States (Getman et al., 2016), Adolescent/Young Adult Medicine and Obstetrics and Gynecology clinics (Trent et al., 2018), 10 different clinical sites (i.e., sexually transmitted disease, family planning, obstetrics-gynecology, and clinical research clinics) in the United States (Sena et al., 2018) were 17.5, 16.3, 16, and 20.5%, respectively. The frequencies of *M. genitalium* in the obstetrics and gynecology clinics in Jalisco, Mexico (Casillas-Vega et al., 2016) and sexually transmitted infection clinics in the Netherlands (de Jong et al., 2016) were only 2.4 and 4.5%, respectively. In addition, a London cohort study discovered that high-risk sexual behavior (more than two sexual partners within 12 months) was an independent risk factor for *M. genitalium* positivity. As the number of sexual partners increased, so did the infection rate. Among women with 0, 1, 2, and 12 partners, the positive rates were 1.2, 2.0, 3.8, and 6.5%, respectively (Oakeshott et al., 2010).

Previous studies and prevalence characteristics indicate that the high-risk factors for *M. genitalium* infection are as follows: black population (Taylor et al., 2018), younger age [age ≤ 30 years (Stafford et al., 2021), age ≤ 25 years (Lillis et al., 2019), age ≤ 20 years (Sena et al., 2018)], symptom status, low socioeconomic status, a series of risky sexual behaviors [increase in a total partner, new partner, and condomless sex, etc. (Taylor et al., 2018). ≥2 sexual partners in the past 12 months (Lillis et al., 2019)]. Due to the low prevalence of *M. genitalium*, universal screening in the general population and pregnant women is not currently supported, but screening cervical secretions from young women with high-risk factors could enhance the diagnosis and treatment of *M. genitalium* infection in women.

**Figure 1:**
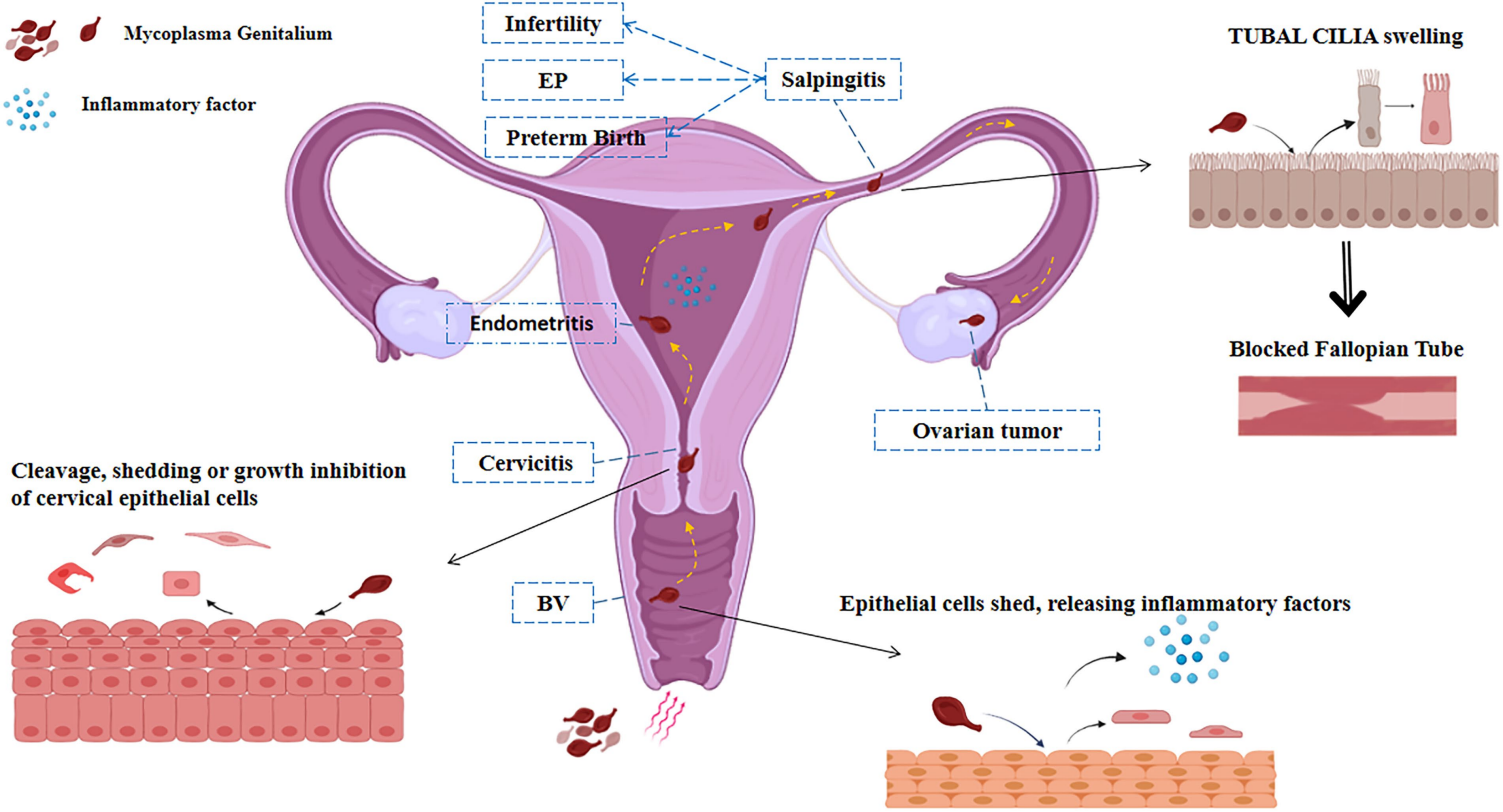
Pathways of infection of *M. genitalium* in the female reproductive system. *M. genitalium* is carried by sperm and other risk factors into the female vagina and colonizes there to cause vaginitis. *M. genitalium* travels up the reproductive tract to the cervix, endometrium, and fallopian tube epithelium, causing cell swelling and shedding, releasing inflammatory factors, and triggering acute inflammatory reactions such as pelvic inflammatory disease and endometritis, inducing adverse pregnancy outcomes such as infertility, preterm delivery, and ectopic pregnancy. Long-term infection with *M. genitalium* transforms into a chronic inflammatory response that may be associated with tumor outcomes such as ovarian cancer.

*Mycoplasma genitalium* infection can cause inflammation of the reproductive system, such as pelvic inflammatory disease, salpingitis, cervicitis, and vaginitis in the female reproductive tract, which may lead to infertility, ectopic pregnancy, and premature delivery, and other reproductive diseases. At the same time, *M. genitalium* may be related to the occurrence and co-infection of ovarian tumors and HPV (Figure 1).

### 3.1. Reproductive tract diseases caused by *Mycoplasma genitalium* infection

#### 3.1.1. *Mycoplasma genitalium* brings about inflammation in the lower genital tract

Inflammation of the lower genital tract includes cervicitis and vaginitis. There is no consensus on cervicitis’s definition and diagnostic criteria. The United States 2021 guidelines for the diagnosis and treatment of sexually transmitted diseases identified two major diagnostic signs that characterize cervicitis: (1) a purulent or mucopurulent endocervical exudate visible in the endocervical canal or on an endocervical swab specimen (commonly referred to as mucopurulent cervicitis) and (2) sustained endocervical bleeding easily induced by gentle passage of a cotton swab through the cervical os (Workowski et al., 2021). *C. trachomatis* and *N. gonorrhoeae* are the two most frequent sexually transmitted pathogens that cause cervicitis, and studies have indicated *M. genitalium* infection as an important new causal pathogen (Roy et al., 2021).

The pathogenic effect of *M. genitalium* on the cervix has been confirmed in the cell (McGowin et al., 2013) and animal experiments (McGowin et al., 2012). Clinical studies have reported that *M. genitalium* infection can cause mucopurulent cervicitis in women (Manhart et al., 2003) and is significantly related to cervical contact bleeding (Oliphant and Azariah, 2013). *In vitro* studies have discovered that 4–14 days after inoculation with high levels of *M. genitalium* (200 MOI), cervical epithelial cells undergo significant cleavage, shedding, or growth inhibition (McGowin et al., 2012), suggesting that *M. genitalium* infection can cause acute toxic reactions in cervical and oviduct epithelial cells, which is directly related to the number of *M. genitalium*. After *M. genitalium* inoculation of cervical epithelial cells for 48 h, interleukin (IL-6, IL-7, IL-8), monocyte chemoattractant protein 1, and granulocyte-macrophage-colony-stimulating factor levels were significantly increased (McGowin et al., 2012, 2013), suggesting that *M. genitalium* infection produces an acute immune response to cervical epithelial cells, leading to increased secretion of inflammatory cytokines. In addition, in the mouse model (McGowin et al., 2010), it was found that *M. genitalium* was detected in the upper genital tract only 3 days after injection of *M. genitalium* into the vagina, suggesting that *M. genitalium* could travel up from the lower end of the genital tract to the upper end. At the same time, the adhesion of *M. genitalium* to cultured cervical epithelial cells *in vitro* can trigger the release of inflammatory signals (Toll-like receptors 2 and 6), which leads to the activation of NF-κB and the induction of host defense-related genes, and ultimately leads to the aggregation of leukocytes at the infection site and the induction of genital tract inflammation (McGowin and Totten, 2017). In contrast, the localization of *M. genitalium* to vaginal and cervical cells provides a survival site and protection from immune responses, as phagocytosis by macrophages can be avoided, thereby facilitating the establishment and maintenance of reproductive tract infections (McGowin et al., 2009). During persistent infection, *M. genitalium* can continuously replicate and provoke the continuous secretion of inflammatory cytokines. *In vitro* studies of cervical epithelial cells have shown that the average number of *M. genitalium* increased 500 to 5,000 times after 85 days of vaccination with a low concentration (10 MOI) of *M. genitalium*. During that time, inflammatory cytokines (IL-6, IL-7, IL-8, monocyte chemoattractant protein 1, and granulocyte-macrophage-colony-stimulating factor) were also detected (McGowin et al., 2012). IL-6 is involved in acute and chronic inflammatory processes and plays a key role in the production of acute phase proteins, the transition from acute to chronic inflammation, and the maintenance of chronic inflammatory response, while weakening the ciliary activity of oviduct epithelium (Papathanasiou et al., 2008).

Likewise, the incidence of cervicitis is significantly higher in *M. genitalium*-positive women than in *M. genitalium*-negative women (Olson et al., 2021), and multiple studies reported *M. genitalium* infection as an independent predictor of cervicitis that increases the risk of cervicitis in women by 2.5–3.3-fold (Lillis et al., 2019). Additionally, *M. genitalium* can cause inflammatory damage to the cervix and endometritis through the cervical canal. The outflow of abnormal cervix secretion also represents a manifestation of inflammation of the upper genital tract.

The current evidence for a link between *M. genitalium* and vaginitis is limited and requires further confirmation. Although *M. genitalium* has been found in vaginal specimens, it is interesting to note whether *M. genitalium* is associated with vaginitis is contentious. Some studies showed a significant correlation between *M. genitalium* infection and bacterial vaginitis (OR, 1.7; Nye et al., 2020; Moore et al., 2021), some showed a negative correlation between *M. genitalium* infection and bacterial vaginitis (OR, 0.4; Manhart et al., 2003), and some showed no correlation between them (*p* > 0.05; Wang et al., 2022). A recent study showed that *M. genitalium* susceptibility might be enhanced in the presence of bacterial vaginitis in a manner similar to *N. gonorrhoeae* and *C. trachomatis* (Shipitsyna et al., 2020). At the same time, there is a strong correlation between *M. genitalium* infection and bacterial vaginosis and trichomonas vaginitis (Masha et al., 2018). Consequently, for women at high risk of *M. genitalium* infection, abnormal vaginal discharge, or cervical contact bleeding, it is necessary to research the detection of *M. genitalium*, timely diagnosis, and effective treatment.

#### 3.1.2. *Mycoplasma genitalium* gives rise to inflammation in the upper genital tract (pelvic inflammatory disease)

Pelvic inflammatory disease describes the inflammation of the upper genital tract, including endometritis, tubal inflammation, tubo-ovarian abscess, pelvic peritonitis, etc., which can present with different clinical symptoms, and some patients present with mild or even unconscious symptoms. In recent years, numerous studies have confirmed that *M. genitalium* is an important new pathogen that causes PID.

*Mycoplasma genitalium* can provoke endometritis and salpingitis in non-human primates (McGowin et al., 2010; Taylor-Robinson and Jensen, 2011), and induce oviduct edema in rats (De Carvalho et al., 2020). Tubal experiments further show that *M. genitalium* can adhere to the surface of cilia, causing abnormal swelling and shedding (Baczynska et al., 2007). Animal studies have revealed that cervical inoculation of *M. genitalium* can infect the uterus and Fallopian tubes (McGowin et al., 2010). Clinical data show that the positive rate of *M. genitalium* infection in endometrial specimens of women with endometritis is 8–12% (Haggerty et al., 2006). Nonetheless, in endometrial and Fallopian tube specimens from patients with acute salpingitis, this proportion is only 4% (Cohen et al., 2005). The detection rate was lower than that of *C. trachomatis* and *N. gonorrhoeae*, which may be related to the mild clinical symptoms of PID caused by *M. genitalium* infection. In addition, the study found that *M. genitalium* infection increased the incidence of PID. Another study reported that the histopathological diagnosis of endometritis incidence was 68.3% in patients with PID and *M. genitalium* positive cervical or endometritis results, but only 44.9% in patients with *M. genitalium* negative results (Haggerty et al., 2008). In addition, a prospective study showed that the incidence of PID in patients with *M. genitalium* infection was 4.9%. In comparison, the incidence of PID in patients with *M. genitalium* negative infection was only 0.6%, suggesting an independent correlation between *M. genitalium* infection and PID (Bjartling et al., 2012). These studies confirm that *M. genitalium* infection can increase the incidence of PID. Previous studies showed that 6 out of 49 women who had *M. genitalium* infection and terminated pregnancy before surgery were infected with PID (incidence: 12.2%), while the infection rate was only 2.4% in 168 women who had no *M. genitalium* infection or *C. trachomatis* infection and terminated pregnancy before surgery (Bjartling et al., 2010). It is suggested that there is a certain relationship between *M. genitalium* infection and PID after the termination of pregnancy. Screening for *M. genitalium* before pregnancy termination and routine treatment of *M. genitalium*-positive patients may prevent PID after pregnancy termination.

Pelvic tenderness is the clinical diagnosis basis for PID, while pelvic inflammation caused by *Mycoplasma genitalium* infection is mild or has no specific discomfort symptoms (Sena et al., 2018), and the changes in inflammatory indicators are not obvious (Ah-Kit et al., 2019). This also leads to infections that are easily overlooked, delayed, or left untreated, leading to long-term reproductive complications, including infertility or ectopic pregnancy (EP) (Ma et al., 2021). Therefore, accurate identification and detection of PID caused by *M. genitalium* infection are crucial, suggesting that PID diagnosis and detection methods need to be further studied.

### 3.2. Reproductive system diseases generated by *Mycoplasma genitalium* infection

#### 3.2.1. *Mycoplasma genitalium* infections may contribute to higher infertility rates

Infertility is defined as the inability to conceive after 12 months of regular unprotected sexual intercourse. Currently, 9% of women of reproductive age worldwide are infertile, among which tubal factors are one of the most common causes of infertility (Carson and Kallen, 2021). *In vitro* and animal experiments have shown that *M. genitalium* infection can cause tubal cilia swelling, shedding (Baczynska et al., 2007), and hydrosalpinx (McGowin et al., 2010). Another study reported 16.1 and 2.2% positivity for *M. genitalium* in cervical swabs from infertile and normal women, respectively (Ajani et al., 2017). This is in agreement with the results of a Polish study on *M. genitalium* (19.9 and 4.4%, respectively; Grzesko et al., 2009), which found that the detection rate of *M. genitalium* in abdominal fluid samples collected by laparoscopy was 5.88 and 0% in infertile and control women, respectively (Grzesko et al., 2009), similar to the results of an Indian study (6.1 and 0.6%; Rekha et al., 2019). The above results suggest that the detection rate of *M. genitalium* in infertile women is significantly higher than that in normal women, which suggests that *M. genitalium* is an independent risk factor for infertility. In 2001, serum levels of antibodies to *M. genitalium* were measured by western blotting in infertile women. The serological positivity rate for MgPa protein of *M. genitalium* was 6.3% in patients with normal Fallopian tubes, and 22.0% in patients with tubal factor infertility (Clausen et al., 2001), which is similar to data from a 2005 study (respectively, 13.2 and 22.0%; Baczynska et al., 2005), were similar. Unfortunately, the experiment only compared rates between groups with infertility factors and not women with childbearing potential.

These findings suggest that the rate of *M. genitalium* infection is higher in cervical and peritoneal fluid and serum from infertile women. This supports previous findings of *M. genitalium* in cervical, endometrial, and Fallopian tube tissue samples, suggesting that *M. genitalium* translocates through the endometrium to the Fallopian tubes, where it attaches to the human Fallopian tube epithelium, impairing tubal function and causing infertility. In addition, *M. genitalium* has been shown to bind to human sperm, with less cell binding at 5 min of incubation; significant binding at 30 min of incubation (Svenstrup et al., 2003). Thus *M. genitalium* may be carried by active sperm to the female reproductive tract and thus be transferred to the uterus and Fallopian tubes, colonizing and destroying the ciliated epithelium *in vivo*, leading to female infertility. These results suggest that routine screening of infertile women for *M. genitalium* and prompt treatment of positive patients may improve infertility outcomes.

#### 3.2.2. *Mycoplasma genitalium* infections and ectopic pregnancy formation

Ectopic pregnancy is a pregnancy implanted outside the intrauterine cavity and accounts for 1–2% of natural pregnancies, whereas more than 98% of pregnancies occur in the Fallopian tube (Ashshi et al., 2015). Salpingitis caused by upper reproductive-tract infection is an important cause of EP (Zhang et al., 2018). *In vitro* experiments involving the oviduct confirmed that *M. genitalium* infection can cause tubal cilia swelling and shedding (Baczynska et al., 2007) and reduce the ciliary activity of the tubal epithelium by increasing IL-6 secretion (Papathanasiou et al., 2008). This suggests that *M. genitalium* promotes EP by changing the structure and function of the Fallopian tubes. Two studies conducted in Saudi Arabia from 2015 to 2016 showed that the rate of *M. genitalium* infection in Fallopian-tube tissues from patients with EP ranged from 19.8 to 20.2%, whereas that in normal Fallopian-tube tissues was only 1.6–3.9% (Ashshi et al., 2015; Refaat et al., 2016). Moreover, they found that serum IL-6 levels in *M. genitalium* infected patients with EP were significantly elevated, suggesting that *M. genitalium* infection was significantly correlated with EP (Ashshi et al., 2015; Refaat et al., 2016).

In 2007, a Swedish study compared serum *M. genitalium* antibodies of pregnant female patients with EP and found an antibody positive rate of 18% in EP patients compared with 15% in controls, indicating no significant correlation between *M. genitalium* antibodies and EP (Jurstrand et al., 2007). Interestingly, another Swedish study in 2015 used the *M. genitalium* serological test to assess differences in *M. genitalium* antibodies between infertile and pregnant women, with the results showing that the rate of *M. genitalium*-positive serum IgG levels in infertile women was 5.4% relative to 1.6% in the control group (Idahl et al., 2015). Furthermore, two other studies reported that patients with a history of PID showed a 7.5-fold increased risk of EP (Ashshi et al., 2015; Refaat et al., 2016). These findings suggest that timely and proper treatment of PID can reduce the incidence of EP.

#### 3.2.3. *Mycoplasma genitalium* infection and preterm birth

Preterm birth is defined as delivery at 28 weeks of gestation but less than 37 weeks, accounting for 5.5–12% of all deliveries, and infectious factors account for 50% of spontaneous preterm births (Kayem et al., 2018). Intrauterine infection is a common and important cause of preterm birth, and epidemiological studies have shown that 25–40% of spontaneous preterm births are associated with intrauterine infections caused by bacteria. *M. genitalium* establishes long-term infection in the upper female genital tract. At the same time, pathogen ascension causes an inflammatory cascade and disrupts the chorionic metaphase gap, which is thought to be the mechanism of genital tract infection leading to preterm labor (Averbach et al., 2013), where the inflammatory factor IL-6 is the main marker of preterm labor. A prospective study on the detection of *M. genitalium* in the vaginal secretions of pregnant women at 23–32 weeks of gestation demonstrated a 20.2% vaginal colonization rate of *M. genitalium* in pregnant women with spontaneous preterm delivery. This suggests that *M. genitalium* colonization is a significant independent risk factor for spontaneous preterm birth (Edwards et al., 2006). Another study of *M. genitalium* testing in spontaneously preterm and term pregnant women within 48 h after delivery showed a 4% (29/661) rate of *M. genitalium* infection in the preterm group and 2% (12/667) in the control group (*p* = 0.007), suggesting that *M. genitalium* positivity is significantly associated with spontaneous preterm delivery (Hitti et al., 2010). An Australian study tested vaginal secretions for *M. genitalium* in the same group of pregnant women at three gestational ages (median gestational age 21, 29, and 36 weeks) and showed a 15 and 2% prevalence of *M. genitalium* infection in women who delivered prematurely and at term, respectively (Payne et al., 2016). However, two prospective studies (Averbach et al., 2013) in pregnant women <11 weeks’ gestation (Kataoka et al., 2006) and < 16 weeks’ gestation reported that vaginal discharge and cervical swab testing for *M. genitalium* showed that the incidence of preterm delivery was not associated with colonization by *M. genitalium*. These data suggest that the median time to clearance of lower genital tract *M. genitalium* infection ranges from 1.5 months to 85.5 days (Lokken et al., 2017; Balkus et al., 2018). Thus, early gestation with *M. genitalium* infection is not the same as late gestation with *M. genitalium* colonization, which is associated with spontaneous preterm delivery in the lower genital tract. This suggests that *M. genitalium* testing should be used as a routine test for microbiology in the lower genital tract in late pregnancy.

In the United States (Kayem et al., 2018) and Australia (Rowlands et al., 2017), the positive rate of amniotic fluid testing for *M. genitalium* in mid-pregnancy was 1.3 and 0%, respectively. In addition, in Italy (Contini et al., 2018) and the United Kingdom (Cox et al., 2016), *M. genitalium* was not detected in chorionic villi samples from spontaneous abortion and placenta after preterm delivery, respectively. These results suggest that *M. genitalium* infections rarely occur in the amniotic cavity, chorionic villi, and placenta.

### 3.3. Co-infection with *Mycoplasma genitalium*

In many countries, *M. genitalium* is generally not recommended for routine sexually transmitted infection (STI) screening due to the lack of information on the prevalence of *M. genitalium* infections and their co-infection with other sexually transmitted bacteria. A study in California showed that the co-infection rate with *M. genitalium* and *C. trachomatis* was 3.1% in women and 9.7% in men (Getman et al., 2016). A study of the prevalence of *C. trachomatis* and *M. genitalium* among women attending termination of pregnancy and contraception clinics during 2009–2019 showed a co-infection rate of 10.1% for *C. trachomatis* and *M. genitalium* (Shilling et al., 2022). In a recent HIV pre-exposure prophylaxis (PrEP) demonstration trial involving 200 men, molecular testing for *N. gonorrhoeae*, *C. trachomatis*, and *M. genitalium* found significantly higher gonococcal bacterial loads in the presence of *M. genitalium*, suggesting that *M. genitalium* may be a cofactor for *N. gonorrhoeae* transmission. If this finding is confirmed in other studies, *M. genitalium* may be required as a cofactor for *N. gonorrhoeae* transmission in specific populations such as MSM (Van Dijck et al., 2022). Therefore, future studies should investigate co-infections with *M. genitalium* and other sexually transmitted bacteria (e.g., *C. trachomatis* or *N. gonorrhoeae*; Desdorf et al., 2021). It is suggested that testing for *M. genitalium* should be included in routine screening for sexually transmitted infections.

Results of a study on the prevalence of *M. genitalium* conducted at the National Sexual Health Clinic in Singapore showed that *M. genitalium* was strongly associated with *C. trachomatis* infection (8.1% of cases) but only 2.4% of *C. trachomatis*-negative cases, suggesting that targeted screening for *M. genitalium* in *C. trachomatis* positive patients could reduce the incidence of *M. genitalium* (Hart et al., 2020). An assessment of the prevalence of *M. genitalium* co-infection in 302 women with *C. trachomatis* attending a clinic for sexually transmitted diseases in Birmingham found only 22 (7.3%) *M. genitalium* co-infections, concluding that *M. genitalium* co-infection is uncommon in women with *C. trachomatis* infection (Harrison et al., 2019). A multicenter clinical study cohort in the United States tested 946 subjects from seven geographically diverse clinical sites for *M. genitalium*, *C. trachomatis*, *N. gonorrhoeae*, and *T. vaginalis*, and found that the prevalence of *M. genitalium* was 16.1% in women and 17.2% in men. The single infection rate of *M. genitalium* in females was significantly higher than the co-infection rate of *C. trachomatis* and *N. gonorrhoeae.* The single infection rate of *M. genitalium* in males was significantly higher than the co-infection rate of *C. trachomatis* and *M. genitalium*, indicating that *M. genitalium* single infection was high. The co-infection rate of *M. genitalium* with other sexually transmitted pathogens was low (Getman et al., 2016). As mentioned above, infection with *C. trachomatis* or *N. gonorrhoeae* is highly likely to be complicated by infection with *M. genitalium*, so the inclusion of *M. genitalium* testing in routine STI screening will likely reduce morbidity.

A 2009 meta-analysis of the association between *M. genitalium* and HIV infection showed a statistically significant twofold increase in the odds of HIV infection (OR 2.1) in the *M. genitalium*-infected population (Napierala Mavedzenge and Weiss, 2009). Since both *M. genitalium* and HIV are sexually transmitted, HIV-positive patients may be more vulnerable to *M. genitalium* infection. A cross-sectional analysis of men who have sex with men (MSM) population in Shenyang, China, found that *M. genitalium* infection (aOR = 3.2) was associated with an increased risk of HIV infection among MSM (Zhao et al., 2019). This suggests the need for more widespread screening for sexually transmitted infections, particularly for *M. genitalium* infections among MSM. However, an African cohort study that examined *M. genitalium* infection in HIV-positive pregnant women by transcription-mediated amplification assay found a higher detection rate of *M. genitalium* in this cohort (21.4%) as well as higher HIV plasma levels in *M. genitalium*-infected women (*p* = 0.02), suggesting that *M. genitalium*-infected women may be more likely to transmit HIV to male sexual partners (Roxby et al., 2019). However, the sample selected for this study was more limited and should be evaluated in a larger study.

The vaginal microbiota plays a role in the acquisition and persistence of HPV in the human vagina and the subsequent development and progression of cervical intraepithelial neoplasia. Noma et al. (2021) found no significant association between *M. genitalium* and high-risk HPV infection. However, this was a statistical investigation of a small sample. A survey of 802 female sex workers showed that *M. genitalium* was significantly associated with certain types of high-risk HPV (HPV-16 and HPV-56) infections (Yin et al., 2013). The results of previous studies on the relationship between *M. genitalium* and high-risk HPV are controversial. In 2018, a meta-analysis showed that *M. genitalium* was associated with a significantly increased risk of high-risk HPV infection (OR 1.50; Ye et al., 2018). Since HPV exposure to cervical intraepithelial neoplasia and cancer progression takes several years, cofactors may play a key role in different stages of the natural history of HPV-induced tumorigenesis. As mentioned above, *M. genitalium* infection can reduce microvilli in cervical epithelial cells, releasing inflammatory factors and disrupting the physical and local immune barriers of the cervix (McGowin et al., 2013). This creates favorable conditions for HPV infection of cervical epithelial basal cells and leads to persistent infection. However, the role of *M. genitalium* as a risk factor for the progression of cervical lesions is unclear and requires further study.

### 3.4. *Mycoplasma genitalium* and reproductive tumor

As early as 1965, Paton et al. (1965) found that *Mycoplasma orale* infection can cause chromosomal abnormalities in cells, which are aggravated with the extension of infection time. In 1966, Macpherson and Russell (1966) demonstrated that some Mycoplasmas act as a “co-carcinogen” and mediate inheritable cell changes *in vitro*. Subsequently, many experiments confirmed that Mycoplasma chronic infection promoted malignant transformation resulting from the dysregulation of genes such as Ras, Myc, or P53 and nuclear factor-κB activation in host cells (Borchsenius et al., 2018). Moreover, Mycoplasma infection promotes tumor progression by increasing the expression of epithelial cell adhesion molecule (Kim et al., 2019), inducing epithelial-mesenchymal transition (Duan et al., 2014b), and activating the β-catenin signaling pathway (Liu et al., 2019) and epidermal growth factor receptor-phosphoinositide 3-kinase-AKT signaling axis (Duan et al., 2014a). Although there is no evidence that Mycoplasma can be directly tumorigenic *in vivo*, analysis of the association between Mycoplasma infection and cell transformation, apoptosis, genomic instability, tumor invasion and metastasis, and drug resistance suggests that Mycoplasma infection is involved in the development of tumors (Li et al., 2022).

The studies on the correlation between mycoplasma and tumor mainly focused on *Mycoplasma hyorhinis*, *Mycoplasma penetrans*, *Mycoplasma fermentans*, and *Mycoplasma hominis*, but very few studies investigated the association between *M. genitalium* and tumors. In 2009, Namiki et al. (2009) first reported the capacity of *M. genitalium* infection to lead to the malignant transformation of benign human prostate cells. A updated meta-analysis showed that a history of PID was associated with an increased risk of ovarian cancer (HR 1.18, 95% CI 1.13 to 1.22; Piao et al., 2020). *M. genitalium* was also linked to ovarian cancer because of its role in PID. A study by Idahl et al. (2011) verified that *M. genitalium* IgG antibodies were associated with borderline ovarian tumors. However, two other studies by Idahl et al. (2020) showed that *M genitalium* is not detectable in ovarian tissues either from women with benign conditions and borderline tumors or from women with ovarian cancer, and serum antibodies to *M. genitalium* were not associated with epithelial ovarian cancers. Fortner et al. (2019) revealed that *M. genitalium* was positively associated with ovarian cancer (response rate: 1.92). However, Trabert et al. (2019) observed no association.

Recent studies indicated that serous ovarian cancer originates in the Fallopian tube and that serous tubal intraepithelial carcinoma represents a precursor lesion in high-grade serous ovarian carcinoma (Wang et al., 2019). As described above, *M. genitalium* typically causes chronic and asymptomatic infection and is associated with PID, tubal pregnancy, and tubal infertility, suggesting that this organism can traverse from the lower to the upper genital tract to cause infection and cause persistent inflammation in the Fallopian tubes and ovaries. Inflammation is characterized by the production of free radicals, cytokines, and prostaglandins. These mediators of inflammation can induce genetic and epigenetic changes, including point mutations in tumor suppressor genes, DNA methylation, and post-translational modifications, causing alterations in critical pathways responsible for maintaining normal cellular homeostasis and leading to the development and progression of cancer (Hussain and Harris, 2007). Accordingly, an association between *M. genitalium* and the risk of ovarian tumors is biologically plausible and can be explained by the inflammation hypothesis.

Currently, epidemiological evidence explaining the potential of *M. genitalium* to cause cancer development is lacking and the possible mechanisms are also unclear. The role of *M. genitalium* in cancer remains conjectural. To improve the understanding of this microorganism’s initial or co-factorial probable roles in cancer, additional *in vitro* and *in vivo* studies on its mechanisms for malignant transformation are needed.

### 3.5. Diagnosis of *Mycoplasma genitalium* infection

The clinical manifestations of *M. genitalium* infection lack specificity, and its diagnosis mainly depends on the etiological diagnosis. Due to the lack of a cell wall, *M. genitalium* is not visible by gram staining microscopy. In addition, *in vitro* culture of *M. genitalium* is difficult and time-consuming, so isolation culture is usually not carried out clinically. And the specificity of the *M. genitalium* serological test is poor, and there is a lack of standard diagnostic reagents. The first *M. genitalium* diagnostic kit was approved by the US Food and Drug Administration (FDA) in January 2019 (Shipitsyna and Unemo, 2020). Due to the widespread presence of macrolide resistance in Europe, the 2021 European Guidelines recommend testing for macrolide resistance mutations in all positive specimens of *M. genitalium* (Jensen et al., 2022). On the types of samples suitable for detection of *M. genitalium*: (1) For men, first void urine (FVU) is usually recommended, which has the advantages of the non-invasive and convenient collection (Gaydos et al., 2019; Van Der Pol et al., 2020); (2) In women, the sensitivity of *M. genitalium* in vaginal swab samples detected by NAAT is higher than that of cervical swabs and urine samples (Gaydos et al., 2019). The United Kingdom guidelines recommend women collect vaginal swab samples (Soni et al., 2019); (3) Rectal samples are recommended in men who have sex with men (MSM) or in patients with proctitis (Jensen et al., 2022).

## 4. Treatment of *Mycoplasma genitalium*-related diseases

Typical therapeutic agents for *M. genitalium* infections are divided into three main groups: macrolides, fluoroquinolones, and tetracyclines. In the past 10 years, the resistance of *M. genitalium* to macrolide antibiotics (azithromycin) as the first-line treatment and to fluoroquinolones (moxifloxacin) as the second-line treatment has increased globally, partly due to the frequent use of similar antibiotics to treat other bacterial pathogens such as *C. trachomatis*, leading to the selection of drug-resistant *M. genitalium* (van der Schalk et al., 2020). The data showed that macrolide resistance for *M. genitalium* increased: from 10% in studies before 2010 to around 50% in studies published in 2016 and 2017, whereas resistance to fluoroquinolones is 7.7% (CI 4.5–11.4; Machalek et al., 2020). And the resistance levels in *M. genitalium* vary by region, population, and gender (Sweeney et al., 2019; Fernandez-Huerta et al., 2020; Iwuji et al., 2022). The increase in drug resistance led to frequent treatment failures, so we need to explore more treatment options and research the resistance mechanisms in *M. genitalium*.

**Figure 2:**
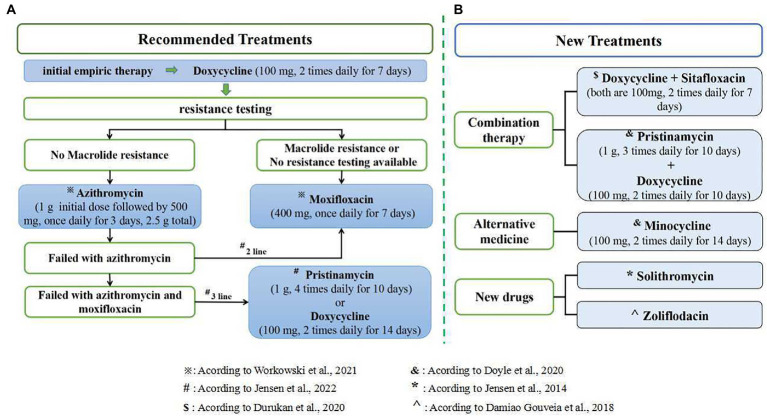
Treatment options for *M. genitalium*. **(A)** The latest recommendations for treatment options following resistance testing; **(B)** several new treatments and drugs related to *M. genitalium* infection.

Resistance-guided therapy is necessary to improve the effectiveness of the treatment for *M. genitalium* infections and reduce macrolide resistance options. Both the 2021 European Guidelines and the 2021 STI Treatment Guidelines recommend that give doxycycline as initial empiric therapy to decrease organism load and the risk of macrolide resistance selection, followed by resistance testing. After the initial 7 days of doxycycline, patients sensitive to macrolides should be given azithromycin, and patients resistant to macrolides should be given moxifloxacin, according to the results of the resistance test (Workowski et al., 2021; Jensen et al., 2022). Sweeney et al. (2019, 2022) highlight the need for ongoing surveillance of resistance in *M. genitalium* and suggest that introducing fluoroquinolone resistance testing would greatly improve the management of *M. genitalium* infection. According to the resistance testing results, the Melbourne Sexual Health Centre gave azithromycin to non-macrolide-resistant patients and sitafloxacin to macrolide-resistant patients, which reached 94.8 and 92.2% of the cure rates, respectively (Read et al., 2019). In 2021 STI Treatment Guidelines, it is recommended that patients with macrolide sensitivity should be given doxycycline followed by azithromycin after screening for macrolide resistance; Patients with macrolide resistance should be given doxycycline followed by moxifloxacin; When resistance testing is not available, patients should be given an initial week of doxycycline followed by a week of moxifloxacin (Workowski et al., 2021). For patients resistant to macrolides and fluoroquinolones, a previous study suggested that pristinamycin is the only clinically available drug to treat *M. genitalium* infections (Martin et al., 2017). This conclusion was supported in an Australian study, which identified bacterial load as an important factor influencing the failure of pristinamycin treatment (Read et al., 2018) and pristinamycin is listed as a third-line treatment in the 2021 European guidelines for the management of *M. genitalium* infections (Jensen et al., 2022). The latest recommendations for treatment options following resistance testing are shown in Figure 2, panel A.

A prospective evaluation (Read et al., 2019) showed that treatment of *M. genitalium* with only a single antibiotic has a high failure rate due to the rapid emergence of resistance. In contrast, combination therapy reduces bacterial escape from antibiotic treatment (Read et al., 2019; van der Schalk et al., 2020). Durukan et al. (2020) found that combination therapy with doxycycline and sitafloxacin was well tolerated and effective in treatment-resistant *M. genitalium*. Meanwhile, Doyle et al. (2020) reported a 75% (55/73) cure rate for macrolide-resistant *M. genitalium* infections treated with a combination of pristinamycin and doxycycline and a 71% (25/35) cure rate with a 14-day course of minocycline. It is suggested that minocycline may be used as an alternative agent for treating *M. genitalium* infections resistant to macrolides and quinolones. However, neither sitafloxacin nor pristinamycin are available in the US at present. In addition, an evaluation of the *in vitro* activity found that fluoroketolide solithromycin has a clinical cure rate of 65 to 85% for the treatment of macrolide-resistant bacteria, suggesting that solithromycin may be a new drug for the treatment of *M. genitalium* infections (Jensen et al., 2014). Damiao Gouveia et al. (2018) found that Zoliflodacin was more effective than moxifloxacin against *M. genitalium* (*p* = 0. 009) through sensitivity evaluation *in vitro*, and there was no cross-reactivity between the two. So Zoliflodacin may also be used as a new drug for the treatment of *M. genitalium* infections. It is suggested that combination therapy can be used, or new drugs and alternative drugs can be developed to reduce drug resistance, shorten the course of treatment, and improve the cure rate. However, a recent prospective review from the Melbourne Sexual Health Centre showed that doxycycline combined with moxifloxacin did not improve treatment in the context of rising quinolone resistance (Vodstrcil et al., 2022). Therefore, the applicability of combination therapy for *M. genitalium* infection needs to be further verified. Several new treatments and drugs for *M. genitalium* infection are shown in Figure 2, panel B.

## 5. Conclusion

*Mycoplasma genitalium* infection is an independent risk factor for female cervicitis and PID. *M. genitalium* can be carried by sperm and other risk factors to the female vagina and colonize there, thus moving up the reproductive tract and causing swelling of the cervix, endometrium, and Fallopian tube epithelium, causing acute inflammatory reactions such as pelvic inflammatory disease and endometritis. Because of the lack of typical clinical manifestations, it is easy to be ignored. Untreated or delayed treatment leads to further transformation of inflammation into chronic long-term infection, resulting in impaired tubal function or blockage, which could cause infertility, ectopic pregnancy, premature birth, and other reproductive diseases. For pregnant women, *M. genitalium* infection can increase the incidence of PID after the termination of pregnancy, and late intravaginal colonization with *M. genitalium* can increase the incidence of preterm delivery. Based on limited studies in recent years, *M. genitalium* may to play a role in the development of ovarian cancer and in the acquisition and persistence of high-risk HPV, but the current findings are inconsistent.

*Mycoplasma genitalium* plays a vital role in inflammatory diseases of the female reproductive tract. However, the question of whether high-risk groups should be screened, how to identify *M. genitalium* infections early, whether timely treatment of *M. genitalium* infections can reduce the incidence of PID, EP, and infertility, and how to improve pregnancy outcomes requires further analysis. Additional epidemiological studies, prospective studies, and *in vitro* and animal studies are needed to confirm whether *M. genitalium* infection can cause tubal inflammation and reduce oncogene activity, leading to ovarian cancer. Whether *M. genitalium* infection increases the risk of high-risk HPV infection and affects the natural history of HPV infection by initiating cellular abnormalities remains to be further explored.

## Author contributions

JY, YZ, and JH prepared and wrote the original draft. HL, XS, TG, and JW provided critical revisions for this review. ZY and ZD contributed to the interpretation and editing of the manuscript. JH is responsible for the concept and final revision of the manuscript. All authors contributed to the review and approved the submitted manuscript.

## Funding

This work was supported by the Scientific Research Project of Hunan Provincial Health Committee (Grant No. 20201915), the Clinical Medical Technology Innovation Guidance Project of Hunan Province (Grant No. 2020SK51901), the Emergency special project of epidemic prevention and control of COVID-19 pneumonia in the University of South China (Grant No. 12), the Hengyang Science and Technology Planning Project (Grant No. 202010021604 and 202250045307), and the Natural Science Foundation of Hunan Province (No. 2022JJ40406).

## Conflict of interest

The authors declare that the research was conducted in the absence of any commercial or financial relationships that could be construed as a potential conflict of interest.

## Publisher’s note

All claims expressed in this article are solely those of the authors and do not necessarily represent those of their affiliated organizations, or those of the publisher, the editors and the reviewers. Any product that may be evaluated in this article, or claim that may be made by its manufacturer, is not guaranteed or endorsed by the publisher.
